# Emergency Department Syndromic Surveillance Diagnostic Code Selection for Assessing Severity of Seasonal Influenza in New South Wales, Australia

**DOI:** 10.1111/irv.70242

**Published:** 2026-03-05

**Authors:** Nectarios Rose, Adam T. Craig, David J. Muscatello

**Affiliations:** ^1^ University of New South Wales Kensington Australia; ^2^ University of Queensland Brisbane Australia

**Keywords:** emergency department syndromic surveillance, ICD‐10, influenza, PISA, SNOMED

## Abstract

**Background:**

Emergency department (ED) syndromic surveillance (EDSyS) often relies on preliminary or ED discharge diagnosis codes as indicators of influenza, but few studies provide a justification for their selection. This retrospective analytical study aimed to optimise the selection of diagnostic codes in EDSyS for monitoring influenza activity and severity.

**Methods:**

Diagnostic codes potentially relating to a respiratory infection and assigned to people presenting to over 180 EDs in New South Wales (NSW), Australia, were grouped into 16 mutually exclusive ‘ED Syndromes’. Time series of the proportion of ED presentations for each ED syndrome by epidemiological week between 2010 and 2019 were compared to a reference series of the percentage influenza positive results from sentinel laboratories, using two similarity and three timeliness statistics. Hospital inpatient admission and laboratory notification data linked to each ED presentation allowed assessment of patient infection status and outcomes.

**Results:**

‘Unspecified Viral’ (any non‐specific viral illness, without reference to the respiratory system) and ED syndromes based on influenza like Illness (ILI) and influenza had the best combination of similarity and timeliness measures. Linked data identified relatively high rates of hospital admission, laboratory‐confirmed influenza and inpatient influenza diagnosis for ED syndromes based on pneumonia and lower respiratory tract infection.

**Conclusion:**

In addition to ILI and influenza, ED syndromes based on unspecified viral illnesses can be used for EDSyS to assess influenza timing and transmissibility in NSW, Australia. The approach outlined in our paper can identify diagnostic codes to improve severity assessment of seasonal influenza using EDSyS.

## 1 Introduction

Worldwide, seasonal influenza is associated with over five million acute lower respiratory associated hospitalisations and approximately 500,000 deaths annually [1]. Given this, and the associated burden influenza has on the health sectors, sensitive and timely surveillance is crucial to identifying the beginning of outbreaks and assessing their severity. Emergency department (ED) syndromic surveillance (EDSyS) systems have been shown to be at least as sensitive to detection of the influenza season's onset as surveillance based on diagnostic data, while offering the added advantage of speed and relative cost‐efficiency [2, 3].

Influenza severity assessment informs risk assessment, public health preparedness response and recovery measures, as well as healthcare resource allocation. The World Health Organization's Pandemic Influenza Severity Assessment (PISA) guidelines describe four dimensions termed ‘indicators’ for seasonal influenza severity assessment; transmissibility, seriousness of disease, morbidity and mortality and impact on health care capacity [4]. PISA recommends parameters related to outpatient visits and laboratory testing to assess transmissibility, with hospital‐based parameters informing the other three indicators. Studies generally use rates of influenza like illness (ILI) presentations or acute respiratory infections (ARI) at GP and outpatient clinics to assess transmissibility. ILI activity in the outpatient and ED settings have been found to correlate closely [5, 6, 7, 8], with studies in Australia [9, 10, 11] and internationally [12, 13] finding that people often presented to EDs for reasons other than disease severity. This suggests that surveillance using ED presentation‐derived data is a reasonable proxy for transmission in the community. As nearly all admissions in Australia related to influenza go through EDs, EDSyS has the potential to provide information on PISA indicators related to hospital outcomes [14], particularly when using integrated electronic medical systems, improving efficiency and reducing inconsistencies that arise from using different data systems [15].

The PISA guidelines recommend that parameters for severity assessment be ‘as indicative of influenza or syndromic respiratory illness activity as possible’. EDSyS usually relies on selecting preliminary or ED discharge diagnosis codes using the International Classification of Disease revision 10 (ICD‐10) [2, 16] to define ARI, severe ARIs (SARI) and ILI, but few studies provide a justification or assess the validity of their selection [17, 18, 19, 20, 21, 22, 23, 24, 25]. Given the potential for EDSyS to inform all indicators of influenza severity, diagnostic code selection should ideally represent diagnoses that capture mild, moderate and severe illness.

Seasonal influenza in NSW, Australia typically occurs between April and October [26, 27]. Using administrative data from EDs across NSW, this retrospective analytical study compares the time series of ED presentations with laboratory‐confirmed influenza positivity to optimise the selection of diagnostic codes for monitoring influenza activity and severity using EDSyS.

## 2 Methods

### 2.1 Data

The study analysed data extracted from the NSW statewide deidentified Pandemic and Epidemic Assessment of Risk Linked data (PEARL) Database. PEARL contains individually linked, anonymised health records of people presenting to over 180 NSW EDs with a condition compatible with ARI for years 2005 through 2023. Linked data included confirmed laboratory‐confirmed influenza notifications as well as hospital inpatient admission records within 28 days of ED presentation [28].

Percentage positive results from sentinel laboratories in NSW by epidemiological week (epiweek) were calculated and used as the reference time series for seasonal influenza activity, representing > 80% of all NSW influenza notifications [29]. Laboratory‐confirmed influenza positivity is available from 2010 onwards [26, 27].

Data from 2020 were omitted due to the disruption caused to seasonal influenza patterns by the COVID‐19 pandemic [30]. Therefore, the study period was 2010 to 2019.

### 2.2 Mapping Diagnosis Codes to ED Syndromes

Only one diagnosis is mandatory in NSW EDs, and diagnoses can represent symptoms or specific conditions. Across NSW, three diagnosis classification systems are used: ICD‐9, ICD‐10 or Systematized Nomenclature of Medicine, Clinical Terminology (SNOMED‐CT) [31]. To consolidate records across systems, codes that indicated influenza or other non‐specific ARI were mapped to 16 categories that we term ‘ED Syndromes’ (Table 1). If a specific organism or diagnosis other than influenza were included in the code description, it was excluded unless the organism is commonly associated with influenza, namely, *Streptococcus pneumoniae* (*S. pneumoniae*), *Staphylococcus aureus* (*S. aureus*) and *Haemophilus influenzae* (*H influenzae*) [32]. A full list of PEARL diagnostic codes and their mapping to ED syndromes are available in Data S1.

**TABLE 1:** Inclusion criteria for including ICD‐9, ICD‐10 and SNOMED codes into ED syndromes.

ED syndrome	Inclusion criteria
Abnormality breathing	Abnormalities in breathing, where infection is not mentioned, e.g., apnoea, asphyxia and orthopnoea
Acute respiratory distress	Acute respiratory failure without specific cause
Bronchiolitis	Bronchiolitis, without reference to a specific organism
Bronchitis	Acute or chronic bronchitis, without reference to a specific organism
Cough	Cough without a specific cause or description indicative of diagnosis
Croup	Croup or symptoms indicative of croup, e.g., barking cough
Fever	Fever or related symptoms and signs without a specific cause, e.g., fever illness and fever of unknown origin
ILI	Specific mention of influenza like illness. SNOMED only category
Influenza	Influenza, including secondary condition caused by influenza
LRTI	Lower respiratory tract infection, respiratory infection or viral pneumonia
URTI	Mention of viral or non‐specific infection of the upper respiratory tract, except sinusitis or bacterial infection
Unspecified viral	Non‐specific viral illness, without reference to the respiratory system
Pneumonia	Pneumonia due to unknown, unspecified bacteria or *S pneumoniae*, *S aureus* and *H influenzae*
Sepsis	Unspecified bacterial sepsis or sepsis caused by *S pneumoniae*, *S aureus* and *H influenzae*
Sinusitis	Sinusitis
Wheezing	Symptoms or signs of wheezing

### 2.3 Analysis

Descriptive analysis was performed, including the temporal distribution of all ED Syndrome presentations by diagnosis classification, and the number of ED presentations and linked hospital admissions for each ED Syndrome. Additionally, we used linked laboratory notification data to calculate the proportion of confirmed influenza cases for each ED syndrome. For ED presentations with a linked inpatient admission, we report the proportion with influenza recorded among the maximum 51 diagnoses available in the hospital admission record.

Counts of ED presentations based on arrival date by epiweek and year from PEARL were divided by the total ED presentations (including non‐ARI presentations) by epiweek to create a time series of rates for each ED Syndrome. These ED Syndrome time series were assessed against the reference time series using similarity and timeliness measures.

Two similarity measures were used to compare ED syndromes to the reference time series after firstly applying a z‐normalisation to each time series [33]: the Pearson correlation coefficient (PCC) and deviation [34] measured using Euclidean distance [35]. To assess the effects of using different diagnosis classification to our similarity results, the PCC was calculated for each ED syndrome with the reference time series by diagnosis classification. As the denominator for ED presentations by diagnosis classification was not available, raw numbers of ED presentations were used instead.

Timeliness—the difference between the time an event occurs and the time the reference standard for that event occurs—was assessed using three measures: peak comparison, aberration detection (start of a season) and the cross‐correlation function (CCF) [36]. The peak of each influenza season was based on the maximum value for each year. For aberration detection, the moving epidemic method (MEM) algorithm developed by Vega et al. [37] was applied using the R function from the MEM package—‘memtiming’ [38]—to identify the start of the influenza season. The mean difference and mean absolute difference between the timing of each ED Syndrome and reference time series were calculated, as was the 95% confidence interval. A negative value for mean difference indicates the ED syndrome timing preceded the reference time series. The CCF was applied to determine the lag period associated with the highest correlation between each ED Syndrome and the reference time series.

Data analysis was carried out using (SAS/STAT) software, Version 9 of the SAS System for Windows. R was used to implement the MEM [39].

## 3 Results

### 3.1 Descriptive Analysis and Similarity Measures

**FIGURE 1:**
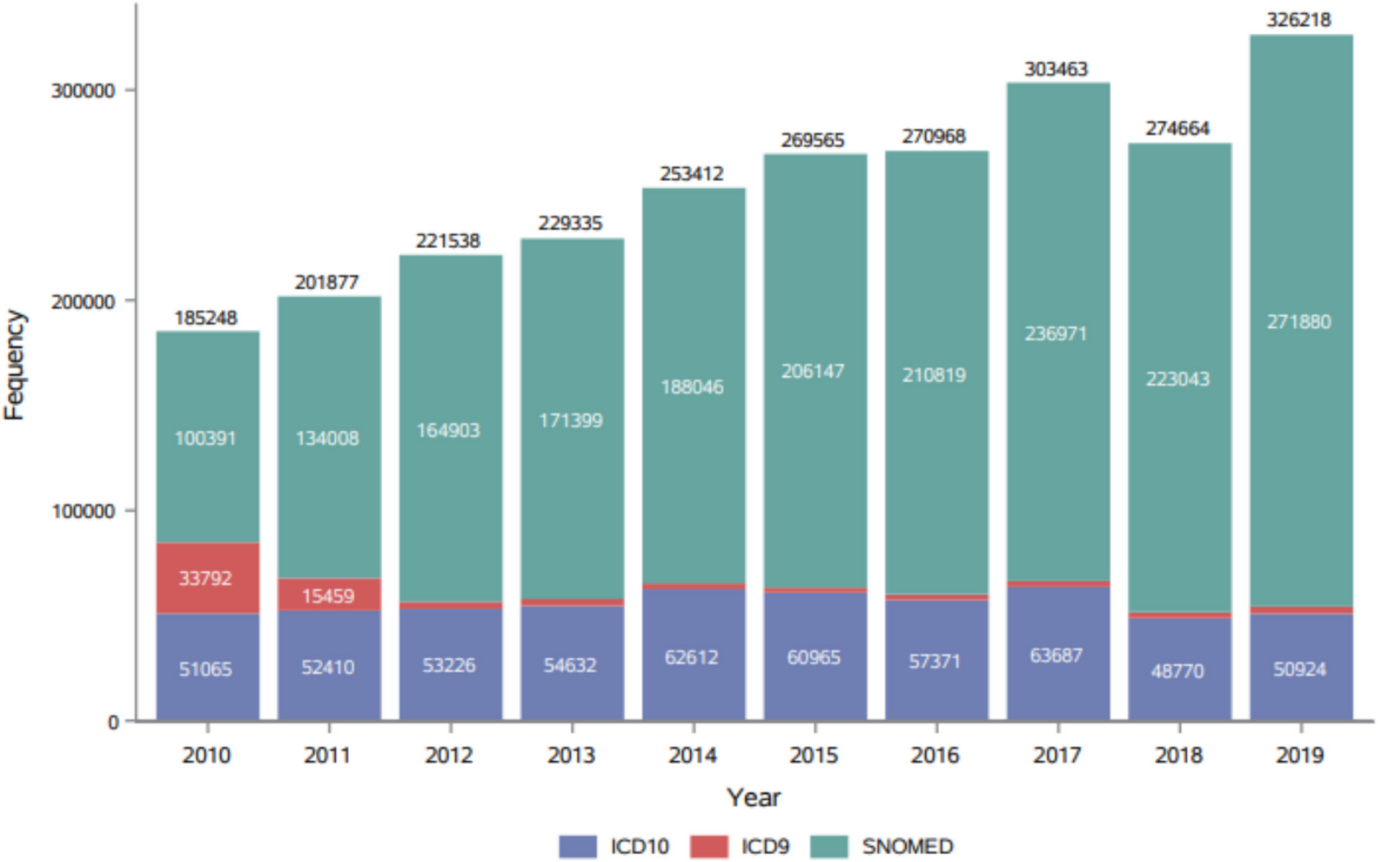
Temporal distribution of clinical diagnosis classification used by included hospitals, 2010–2019.

Over the study period, there were 4,134,100 ED presentations meeting the inclusion criteria, in which 34% linked to an admission and 2.2% linked to a positive laboratory result. All ED Syndromes had a statistically significant PCC (*p* < 0.05), with Unspecified Viral having the highest of 0.88 (Table 2). By 2012, most hospitals had switched to the SNOMED diagnosis classification system, with only one hospital using ICD‐9 (Figure 1).

**TABLE 2:** Similarity and other measures for each ED syndrome against the reference influenza series.

ED syndrome	PCC	Euclidean distance	ED count	Admission rate	Lab confirmed rate	Inpatient influenza diagnosis rate
Unspecified viral	0.88	5.6	609,487	16%	2%	4%
ILI	0.78	8.6	39,119	11%	10%	28%
URTI	0.78	8.3	1,095,451	14%	1%	2%
Influenza	0.77	9.1	65,297	24%	42%	64%
LRTI	0.74	8.5	281,878	41%	3%	4%
Pneumonia	0.73	9.2	374,440	79%	3%	3%
Cough	0.63	11.1	208,122	17%	2%	3%
Sinusitis	0.57	14.2	45,036	14%	1%	1%
Acute Respiratory distress	0.54	15.3	23,685	84%	3%	2%
Abnormality breathing	0.53	13.4	328,157	59%	1%	2%
Bronchitis	0.51	10.4	66,145	22%	1%	2%
Pleurisy	0.45	15.3	19,969	21%	0%	1%
Fever	0.38	14.6	422,010	49%	2%	3%
Bronchiolitis	0.33	12.4	168,687	56%	1%	2%
Sepsis	0.23	15.0	142,542	97%	2%	1%
Croup	0.16	12.2	244,075	14%	1%	1%

**FIGURE 2:**
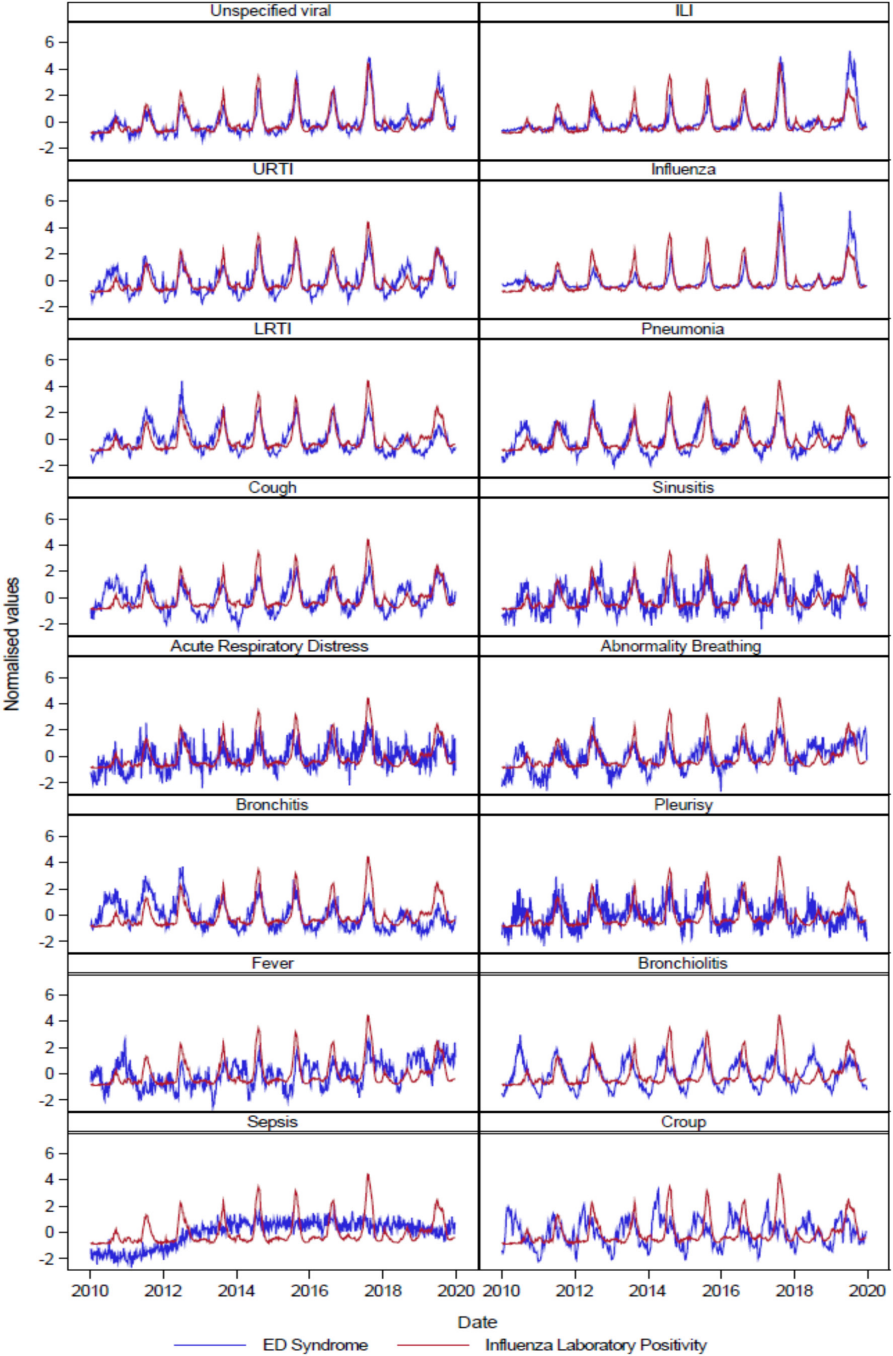
Normalised time series of each ED syndrome and laboratory‐confirmed influenza percentage positive.

In Figure 2, each of these normalised ED Syndromes is shown with the reference time series on the same axis. Visually, ‘Unspecified viral’, ILI, LRTI and Influenza are most like the reference time series in terms of the timing and magnitude of seasonal peaks and interseasonal activity, in contrast to Pleurisy, Sepsis, Croup and Fever.

Examination of ICD10 and SNOMED PCC values and their 95% confidence intervals shows there is no significant difference between the diagnosis classification estimates for Unspecified Viral, Influenza and Pneumonia compared to the reference time series (Table 3). There is however a significant difference for LRTI.

**TABLE 3:** Pearsons's correlation coefficient of selected ED syndromes (based on counts) relative to the laboratory‐confirmed influenza percentage positive time series by diagnosis classification with 95% confidence intervals. ICD‐9 based presentations were excluded due to very small numbers.

ED syndrome	Overall	ICD10	SNOMED
Unspecified viral	0.81 (0.78 to 0.84)	0.75 (0.71 to 0.78)	0.77 (0.74 to 0.81)
ILI	0.77 (0.72 to 0.79)	NA	0.76 (0.72 to 0.79)
URTI	0.77 (0.73 to 0.80)	0.63 (0.57 to 0.68)	0.69 (0.64 to 0.73)
Influenza	0.75 (0.71 to 0.79)	0.74 (0.70 to 0.78)	0.73 (0.69 to 0.77)
LRTI	0.83 (0.80 to 0.86)	0.71 (0.67 to 0.75)	0.83 (0.80 to 0.85)
Pneumonia	0.78 (0.74 to 0.81)	0.71 (0.67 to 0.75)	0.74 (0.70 to 0.78)
Cough	0.69 (0.64 to 0.73)	0.51 (0.44 to 0.57)	0.66 (0.61 to 0.71)
Sinusitis	0.58 (0.53 to 0.64)	0.35 (0.27 to 0.42)	0.57 (0.51 to 0.62)
Acute respiratory distress	0.57 (0.50 to 0.62)	0.26 (0.16 to 0.34)	0.52 (0.46 to 0.58)
Abnormality breathing	0.49 (0.42 to 0.55)	0.57 (0.51 to 0.63)	0.40 (0.32 to 0.47)
Bronchitis	0.66 (0.60 to 0.70)	0.44 (0.37 to 0.51)	0.71 (0.67 to 0.75)
Pleurisy	0.54 (0.47 to 0.60)	0.356 (0.28 to 0.43)	0.50 (0.43 to 0.56)
Fever	0.37 (0.30 to 0.45)	0.28 (0.20 to 0.36)	0.34 (0.26 to 0.42)
Bronchiolitis	0.40 (0.33 to 0.47)	0.35 (0.27 to 0.42)	0.4 (0.336 to 0.4)
Sepsis	0.25 (0.17 to 0.33)	0.16 (0.08 to 0.25)	0.27 (0.18 to 0.343)
Croup	0.25 (0.16 to 0.33)	0.19 (0.10 to 0.27)	0.29 (0.21 to 0.37)

### 3.2 Timeliness Measures

The LRTI ED Syndrome had a mean absolute difference for peaks of less than 1 week (Table 4), with Pneumonia, Unspecified Viral, Influenza and ILI having a mean absolute difference of less than 2 weeks. In contrast, the mean absolute difference for the start of the influenza season was greater than 1 week for all indicators, with Influenza having the smallest mean absolute difference of 1.9 weeks followed by Unspecified Viral with 2.4 weeks (Table 5).

**TABLE 4:** Mean difference and mean absolute difference in weeks between peaks for each year of time series for each ED syndrome compared laboratory‐confirmed influenza percentage positive. ED syndromes with a mean absolute difference of 5 or less are shown and are sorted by increasing mean absolute difference.

ED Syndrome	Mean difference in weeks (95% CI)	Mean absolute difference in weeks (95% CI)
LRTI	−0.1 (−1.1 to 0.9)	0.9 (0.2 to 1.6)
Pneumonia	0.2 (−1.0 to 1.4)	1.2 (0.5 to 1.9)
Unspecified viral	1.2 (0.1 to 2.3)	1.2 (0.1 to 2.3)
Influenza	−0.1 (−1.4 to 1.2)	1.3 (0.5 to 2.1)
ILI	0.2 (−1.6 to 2.0)	1.8 (0.6 to 3.0)
Cough	0.4 (−3.5 to 4.3)	2.8 (−0.5 to 6.1)
Bronchitis	0.8 (−3.0 to 4.6)	3.0 (−0.1 to 6.1)
URTI	3.5 (−2.4 to 9.4)	4.1 (−1.6 to 9.8)
Pleurisy	−3.2 (−7.8 to 1.4)	4.6 (0.7 to 8.5)
Acute respiratory distress	−2.0 (−6.7 to 2.7)	4.8 (1.5 to 8.1)

**TABLE 5:** Mean difference and mean absolute difference in weeks between the start for each year of time series for each ED syndrome compared laboratory‐confirmed influenza percentage positive as identified by the moving epidemic method. ED syndromes with a mean absolute difference of 5 or less are shown and are sorted by increasing mean absolute difference.

ED syndrome	Mean difference in weeks (95% CI)	Mean absolute difference in weeks (95% CI)
Influenza	−0.5 (−2.1 to 1.1)	1.9 (1.0 to 2.8)
Unspecified viral	−1.0 (−3.1 to 1.1)	2.4 (1.2 to 3.6)
Acute respiratory distress	0.0 (−2.4 to 2.4)	2.4 (0.8 to 4.0)
ILI	−1.8 (−4.3 to 0.7)	2.6 (0.5 to 4.7)
URTI	−3.3 (−5.4 to −1.2)	3.7 (2.0 to 5.4)
LRTI	−3.5 (−5.8 to −1.2)	4.1 (2.4 to 5.8)
Fever	−3.2 (−7.9 to 1.5)	4.4 (0.3 to 8.5)

Analysis of the CCF found that ILI and Influenza had higher correlations with a lag of 2 weeks relative to the reference series, while Unspecified Viral lagged 1 week (Table 6).

**TABLE 6:** Lag in weeks of each ED syndrome having the highest Pearson's correlation coefficient (PCC) value relative to laboratory‐confirmed influenza percentage positive values (reference time series). Only ED syndromes with an optimal lag within 5 weeks of the reference time series are shown.

ED syndrome	PCC	Lag (weeks)
	0.91	1
ILI	0.82	2
URTI	0.78	0
Influenza	0.82	2
LRTI	0.74	0
Pneumonia	0.73	0
Cough	0.63	0
Sinusitis	0.58	1
Acute respiratory distress	0.54	0
Abnormality breathing	0.55	−3
Bronchitis	0.51	−1
Pleurisy	0.46	−2
Fever	0.42	2

## 4 Discussion

This paper applies an approach to selecting and validating diagnostic codes for EDSyS that can be used to inform indicators measuring the severity of seasonal influenza. Percentage positivity of laboratory‐confirmed influenza is often used as a measure of transmissibility [4] and the combination of high similarity and timing measures for the ED Syndromes—Unspecified Viral, ILI and Influenza—suggests they are suitable for assessing seasonal influenza transmissibility in NSW, Australia, with the advantage of more timely results and more complete regional representation. These ED Syndromes were also the best for identifying the start of the influenza season using the MEM.

It is surprising that the ED Syndrome—Unspecified Viral—scored higher similarity measures than the more specific ED Syndromes based on an influenza diagnosis. Unspecified Viral is based on ICD and SNOMED codes that describe viraemia or other non‐specific viral infections. Similar ICD‐10 codes—in particular B34, ‘Viral infection’—have been included in other studies for the syndromic surveillance of influenza activity [17, 41, 42, 43, 44]. One explanation for these high similarity and timing metrics is that the ED Syndrome—Unspecified Viral—represents influenza cases that were not diagnosed at the time of ED presentation. The low hospital influenza diagnosis rate for Unspecified Viral cases (4.0%) may be in part a result of low testing practices [44] and the omission by hospital administrative coding staff in assigning a code for influenza [46, 47].

Fever, in combination with cough or sore throat, is often used as an indicator for influenza [4, 5, 6, 8, 48]; however, only a single diagnosis or chief complaint can be assigned for each ED presentation in most EDSyS systems [2, 16]. A recent study used fever only (measured or self‐reported) in EDSyS to forecast influenza hospital visits, arguing it to be a more specific and objective symptom than cough or sore throat [48]. The use of antipyretics or reliance on an oral history, however, can lead to inconsistencies in how fever is assigned [49]. Indeed, in our study, the ED Syndrome, Fever, had a PCC of only 0.38, while Cough had a much higher PCC of 0.65. Despite the relatively high PCC, Cough performed poorly on timeliness measures. Based on our results, we would not recommend the use of fever or cough related diagnostic codes for EDSyS systems where only a single diagnosis or chief complaint is captured.

The use of diagnostic codes to assess correlation with laboratory‐confirmed influenza diagnosis has been performed before, although our study also included timing metrics. This is the first study to our knowledge to assess the use of SNOMED codes in the context of EDSyS. A study carried out in two ED Departments in Victoria, Australia [40], found ‘J11 Influenza virus not identified’, ‘J06 Acute upper respiratory infection multiple and unspecified sites’, ‘J22 Unspecified acute lower respiratory infection’, ‘B34 Viral infection, unspecified site’ and ‘J18 Pneumonia organism unspecified’ to each have a Spearman's correlation of greater than 0.3 (*p* < 0.05), and a combined correlation of 0.56. In contrast, our paper uses ICD‐10 codes grouped into logical ED Syndromes, pooling a greater number of cases. This is important when using SNOMED, where mapping rules from ICD‐10 to over 100,000 SNOMED codes are not always accurate [50]. The pooling of diagnoses into ED syndromes and use of NSW wide ED presentation may explain why our study achieved higher PCCs, particularly when combining all syndromes where an PCC of 0.83 was found.

Most of our ED Syndrome time series had poor timing measures for the start of the influenza season. Aberration detection for the start of a season is often based on a threshold [51], scan statistics [52], cumulative sum [44, 54] or exponential weighted moving average [54]. These methods rely on parameter and target values, usually derived by using information from past seasons [36]. We applied the widely used MEM to identify the onset of influenza outbreaks [22, 37, 48, 56, 57] as it includes a step that does not rely on previous season data to identify the start of a season [37]. The MEM results are supported by a visual comparison of ED Syndrome and reference time series, which show that only Influenza, Unspecified Viral and ILI have a clear upswing in values coinciding with the start of a season. While LRTI and Pneumonia do show poor timing for the start of the influenza season, they perform the best in regard to the timing of the peak of a season. Given the high rates of admission for these ED syndromes (Table 2), LRTI and Pneumonia may be used for PISA indicators related to hospitalisation and severe outcomes. In NSW, the EDSyS system known as PHREDSS [58] uses diagnostic codes based on ILI and influenza to identify the start of the season and monitor hospital admissions as an indicator of severity [27]. Our results suggest that PHREDSS could expand its diagnostic code criteria to include Unspecified Viral related codes for monitoring transmissibility, and diagnostic codes related to LRTI and Pneumonia to improve assessment of hospital related indicators of seasonal severity.

A limitation of this study is that the definition of ED Syndromes may be considered arbitrary. Although the identification of codes for conditions such as ‘Unspecified Viral’ and Influenza (See Supporting Information) is straightforward, other ED Syndromes such as URTI and LRTI pose challenges due to the heterogeneity of the diagnostic codes used. This may explain the differences in PCCs seen between ICD‐10 and SNOMED codes for these conditions. While the selection and performance of the ED Syndromes are specific to NSW, Australia, the method can be applied to any region where EDSyS and an appropriate reference time series are available.

## 5 Conclusion

This study outlines the use of time series similarity and timing measures for selecting diagnostic codes as indicators for EDSyS seasonal influenza severity assessment. In addition to ILI and influenza, our approach identified diagnoses based on unspecified viral illnesses for assessing influenza timing and transmissibility in NSW, Australia, as well as pneumonia and lower respiratory tract infections for assessing hospital‐related severity outcomes.

## Author Contributions

**Nectarios Rose:** conceptualisation, data curation, formal analysis, methodology, software, validation, visualisation, writing – original draft, writing – review and editing. **Adam T. Craig:** supervision, writing – review and editing. **David J. Muscatello:** funding acquisition, supervision, writing – review and editing.

## Conflicts of Interest

The authors declare no conflicts of interest.

## Supporting information


**Data S1:** Supporting information.

## Data Availability

The data that support the findings of this study are available from The Centre for Health Record Linkage (CHeReL). Restrictions apply to the availability of these data, which were used under license for this study. Data are available from https://www.cherel.org.au/with the permission of the New South Wales Ministry of Health.
